# Evolving the Whale Optimization Algorithm: The Development and Analysis of MISWOA

**DOI:** 10.3390/biomimetics9100639

**Published:** 2024-10-18

**Authors:** Chunfang Li, Yuqi Yao, Mingyi Jiang, Xinming Zhang, Linsen Song, Yiwen Zhang, Baoyan Zhao, Jingru Liu, Zhenglei Yu, Xinyang Du, Shouxin Ruan

**Affiliations:** 1 School of Mechanical and Electrical Engineering, Changchun University of Science and Technology, Changchun 130022, China; licf@cust.edu.cn (C.L.); yaoyq@mails.cust.edu.cn (Y.Y.); zhyw@cust.edu.cn (Y.Z.); 2 School of Mechatronic Engineering and Automation, Foshan University, Foshan 528225, China; 3 The People’s Liberation Army (PLA) Unit 63850 of China, Changchun 130022, China; jiang_ming_yi@126.com; 4 FAW Tooling Die Manufacturing Co., Ltd., Changchun 130013, China; zhaobaoyan@faw.com.cn (B.Z.); liuqr_td@faw.com.cn (J.L.); duxinyang_td@faw.com.cn (X.D.); 5 College of Biological and Agricultural Engineering, Jilin University, Changchun 130022, China; zlyu@jlu.edu.cn; 6 China FAW Group Corporation, Changchun 130000, China; ruanshouxin@faw.com.cn

**Keywords:** Whale Optimization Algorithm, Multi-Swarm Optimization, adaptive spiral indentation strategy, global search capability, optimization algorithm robustness

## Abstract

This paper introduces an enhanced Whale Optimization Algorithm, named the Multi-Swarm Improved Spiral Whale Optimization Algorithm (MISWOA), designed to address the shortcomings of the traditional Whale Optimization Algorithm (WOA) in terms of global search capability and convergence velocity. The MISWOA combines an adaptive nonlinear convergence factor with a variable gain compensation mechanism, adaptive weights, and an advanced spiral convergence strategy, resulting in a significant enhancement in the algorithm’s global search capability, convergence velocity, and precision. Moreover, MISWOA incorporates a multi-population mechanism, further bolstering the algorithm’s efficiency and robustness. Ultimately, an extensive validation of MISWOA through “simulation + experimentation” approaches has been conducted, demonstrating that MISWOA surpasses other algorithms and the Whale Optimization Algorithm (WOA) and its variants in terms of convergence accuracy and algorithmic efficiency. This validates the effectiveness of the improvement method and the exceptional performance of MISWOA, while also highlighting its substantial potential for application in practical engineering scenarios. This study not only presents an improved optimization algorithm but also constructs a systematic framework for analysis and research, offering novel insights for the comprehension and refinement of swarm intelligence algorithms.

## 1. Introduction

Meta-heuristic algorithms have demonstrated remarkable efficacy in tackling the NP puzzle by adeptly navigating the minimization or maximization of objective functions within a constrained response time framework [1,2,3,4,5]. These algorithms have been instrumental in advancing the field of computational intelligence. Swarm intelligence (SI) algorithms, drawing inspiration from the collective behaviors of animal or insect groups that exhibit coordinated action and environmental responsiveness, have emerged as a significant paradigm within this domain. The adaptability, operational simplicity, and inherent reliability of SI algorithms [6] have garnered considerable attention and have propelled their integration into a myriad of applications. Highly cited pioneering and recently proposed SI algorithms include Ant Colony Optimization (ACO) [7], Particle Swarm Optimization (PSO) [8], the Cuckoo Optimization Algorithm (COA) [9], Gray Wolf Optimization (GWO) [10], and the Whale Optimization Algorithm (WOA) [11]. SI algorithms are distinguished by their uniform structure, simplicity, adaptability, robustness, rapid convergence, and a smaller number of parameters [12,13]. Consequently, a variety of SI algorithms have been crafted to address optimization challenges across diverse fields, including medical and engineering applications. Nonetheless, SI algorithms encounter several limitations. An inferior search strategy [14] leads to premature convergence [15], imbalance issues [16], entrapment in local optima [17], and reduced population diversity [18,19].

In 2016, Mirjalili and Lewis introduced the Whale Optimization Algorithm (WOA) [11], an innovative metaheuristic inspired by the sophisticated foraging strategies of humpback whales. The WOA’s principal allure is its simplicity coupled with an efficient search mechanism, which facilitates the rapid identification of optimal solutions. Despite its merits, the WOA, like other population-based intelligence algorithms, is not impervious to challenges. It can encounter issues such as entrapment in local optima, premature convergence, and diminished population diversity. Jianxun Liu et al. [20] proposed a novel Enhanced Global Search Whale Optimization Algorithm (EGE-WOA) in order to improve the convergence behavior of the WOA and enhance its global search capability. Juan Du et al. [21] proposed an improved whale optimization algorithm for time-optimal trajectory planning in response to the problems of unstable, uneven, and inefficient motion trajectories of traditional manipulator systems. Lisang Liu et al. [22] proposed the second-order evolutionary chaotic whale optimization algorithm (DECWOA) to address the shortcomings of the WOA’s insufficient global search capability and slow convergence speed; using various randomly selected test functions, they analyzed the impact of various improvement strategies on the algorithm’s performance. Chao-Hsien Hsieh et al. [23] proposed an enhanced whale optimization algorithm with chaotic mapping and an adaptive iteration strategy (CMAISWOA) and evaluated the efficacy of the proposed CMAIS-WOA using 13 classical benchmark functions and the IEEE CEC2014 test suite. A multitude of enhanced WOA algorithms have been proposed, yet the majority of these enhancements fail to acknowledge the impact of the spiral updating method on the algorithm’s optimization search and the algorithm’s lack of global search capability. Conversely, there is no consideration or compensation for the algorithm’s diminished arithmetic capabilities in one aspect due to the addition of weights.

Facing the growing complexity of optimization problems, traditional algorithms often struggle to meet the demands for efficient and precise solutions. Swarm intelligence algorithms, with their exceptional ability to simulate the collective behaviors of biological groups in nature, offer innovative approaches to these challenges. However, existing algorithms, including the Whale Optimization Algorithm (WOA), still encounter performance bottlenecks, such as a propensity to become trapped in local optima, slow convergence rates, and insufficient population diversity. Consequently, enhancing these algorithms to bolster their global search capabilities and convergence performance holds significant theoretical and practical implications for advancing the field of optimization algorithms. This study is dedicated to the enhancement of the Whale Optimization Algorithm (WOA) through the proposition of an innovative Multi-Swarm Improved Spiral Whale Optimization Algorithm (MISWOA). This research undertakes a comprehensive examination of the WOA, meticulously identifying its limitations pertaining to global exploration and local convergence capabilities. Subsequently, it delves into the development of pertinent strategic improvements aimed at addressing these shortcomings.

The main work of this paper includes the following:

- Enhanced Convergence Factor: This paper introduces a nonlinear convergence factor augmented with an adaptive compensation mechanism, designed to enhance the algorithm’s search capability in its later stages, thereby improving the algorithm’s search efficiency and convergence accuracy.
- Adaptive Weight Design: The study proposes a framework incorporating a variable gain weighting mechanism that dynamically recalibrates the algorithm’s search strategy throughout the entire search process. By balancing the algorithm’s search and convergence capabilities, it enhances the overall performance and robustness of the algorithm.
- Improved Spiral Convergence Method: The study pioneers the enhancement of the spiral structure by integrating a novel spiral shape coefficient. This improvement allows the algorithm to flexibly adjust its spiral convergence method when faced with search spaces of arbitrary dimensions, thereby endowing the algorithm with higher intelligence and adaptability.

Multi-Swarm Collaboration Strategy: This study establishes a multi-population mechanism to simulate the collaborative dynamics of whale pods in nature, thereby enhancing the intelligence of the algorithm. In this improvement, whales are assigned to two sub-populations based on “Task codes” at the time of generation, which may have a negative impact on search and convergence in some cases. However, this enhancement helps the population to consistently find optimal solutions in the majority of situations. Through task collaboration among multiple populations, the whale pod enhances the overall efficiency and robustness of the algorithm through a task-coordinated approach.

The “Simulation + Experimentation” Validation Approach: This manuscript employs a “simulation + experimentation” methodology to ascertain the efficacy of the enhanced algorithm. By conducting comparative analyses with algorithms from the iterative improvement process, this study facilitates both analysis and refinement, culminating in the introduction of a Multi-Swarm Improved Spiral Whale Optimization Algorithm (MISWOA). Through comparative assessments with the original Whale Optimization Algorithm (WOA), its derivatives, and other distinguished algorithms, the validation of the improvement methodology’s effectiveness and the exemplary performance of MISWOA is established. Ultimately, empirical testing is conducted to further corroborate the efficacy of the algorithmic enhancements and to highlight the significant potential of MISWOA in real-world engineering scenarios.

This manuscript introduces the Multi-Swarm Improved Spiral Whale Optimization Algorithm (MISWOA), which has undergone extensive experimental validation across a spectrum of standard test functions. The results demonstrate that the MISWOA outperforms the foundational Whale Optimization Algorithm (WOA) in terms of search accuracy and convergence velocity, effectively mitigating the issues of premature convergence and entrapment in local optima. The paper contributes not only an enhanced optimization algorithm but also constructs a systematic framework for analysis and research. This framework offers novel perspectives for understanding and refining swarm intelligence algorithms, enriching the academic discourse in the field.

## 2. Enhancement Strategies for the Whale Optimization Algorithm

The Whale Optimization Algorithm (WOA) is meticulously crafted to replicate the sophisticated predatory tactics exhibited during the coordinated hunts of humpback whales. This bio-inspired computational strategy encapsulates three distinct phases of the whales’ hunting behavior: the strategic encirclement of prey, the deployment of a bubble net for entrapment, and the comprehensive search for prospective targets.

**Figure 1:**
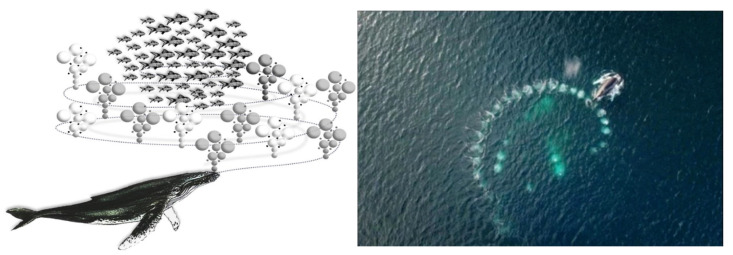
Whale roundup process.

The predation process, as illustrated in Figure 1, commences with the whale executing a spiral descent from the seabed, emitting a profusion of bubbles in diverse dimensions. These bubbles rise buoyantly toward the water’s surface, amassing to create an enveloping, cylindrical mesh that encapsulates the intended quarry. In a strategic ascent, the whale spirals upward, incrementally constricting the parameters of the encirclement. This deliberate motion culminates in the whale’s arrival at the precise coordinates of the targeted fish school, where it engages in foraging activities.

### 2.1. Mathematical Framework: Modeling Predatory Behaviors in the WOA

- Enveloping the prey.

The formula for updating the position of the whale in this process is as follows:

(1) $$\left\{\begin{array}{c} X\left(t+1\right)=X^{*}\left(t\right)-A\cdot D\\ D=\left|C\cdot X^{*}\left(t\right)-X\left(t\right)\right| \end{array}\right.$$

In Equation (1), *D* is the distance vector between the humpback whale and its prey, and *t* is the number of iterations; *X* is the individual position; X* is the global optimal position; *A* and *C* are the coefficient matrices.

(2) $$\left\{\begin{array}{c} A=2ar_{1}-a\\ a=2-\frac{2t}{t_{\max}}\\ C=2r_{2} \end{array}\right.$$

In Equation (2), *t* and $t_{max}$ are the number of iterations and the maximum number of iterations, respectively; $r_{1}$ and $r_{2}$ are both random numbers within [0,1]; and a is the convergence factor, which decreases linearly from 2 to 0.

- Predation by bubble netting.

The humpback whale ascends in a spiral motion, expelling a network of bubbles in order to ensnare its prey in a circular formation. The process is described by the following expression:

(3) $$\left\{\begin{array}{c} X\left(t+1\right)=X^{*}\left(t\right)+D\cdot e^{bl}\cdot \cos\left(2\pi l\right)\\ D=\left|C\cdot X^{*}\left(t\right)-X\left(t\right)\right| \end{array}\right.$$

In the expression: b is a constant that changes the shape of the spiral and l is a random number within [0,1].

During the predation of humpback whales, the two processes of encirclement and attack occur simultaneously. Consequently, the updated expression is:

(4) $$\left\{\begin{array}{c} X\left(t+1\right)=X^{*}\left(t\right)-A\cdot D,p<0.5\\ X\left(t+1\right)=X^{*}\left(t\right)+D\cdot e^{bl}\cdot \cos\left(2\pi l\right),p\geq 0.5 \end{array}\right.$$

where *p* is a random number within [0,1].

- Random search.

In addition to the processes described above, humpbacks simultaneously search for prey in areas outside the target. To mimic such behavior, subsequent actions are judged based on a variable coefficient A: if $\left|A\right|\geq 1$, the whale is outside the perimeter and chooses to search randomly; if $\left|A\right|<1$, the whale is inside the perimeter and chooses to join the attack. The update formula for the random search is:

(5) $$\left\{\begin{array}{c} X\left(t+1\right)=X_{rand}\left(t\right)-A\cdot D\\ D=\left|C\cdot X_{rand}\left(t\right)-X\left(t\right)\right| \end{array}\right.$$

In the update formula, $X_{rand}$ is the position of one random whale. The above three components together form the whale optimization algorithm.

### 2.2. Algorithmic Refinement: Enhancing the WOA’s Performance Metrics

#### 2.2.1. Performance Analysis: Dissecting the WOA’s Mathematical Model

The foundational Whale Optimization Algorithm (WOA) undergoes a preliminary evaluation through its integration with a comprehensive suite of six standardized test functions, designed to rigorously probe its optimization capabilities. In this examination, both the foundational Whale Optimization Algorithm (WOA) and the subsequently enhanced versions of the WOA have been configured with uniform parameter settings. The population size of the WOA and the improved WOA presented in subsequent chapters is set to 50, and the number of iterations is set to 500. The functional expressions and parameter settings of the test functions are shown in the following Table 1.

The operating environment of the simulation experiments in this article is a Core i7 CPU, Windows 10 operating system, and a dual-core Inter TM. The physical memory is 16 GB, and the speed of the processor is 1 GHz. The algorithm is run on MATLAB R2021a software. The subsequent assessment outcomes, which offer insights into the algorithm’s performance, are presented in a structured manner below.

The selection of test functions is strategically designed to evaluate various aspects of algorithmic performance. Test functions 1 and 2 are meticulously crafted to measure the convergence velocity and solution precision of the algorithm. Functions 3 and 4 are engineered to scrutinize the algorithm’s proficiency in global search endeavors. Furthermore, test functions 5 and 6 are formulated to appraise the algorithm’s capacity to evade entrapment in local optima and navigate towards the global optimum.

**Figure 2:**
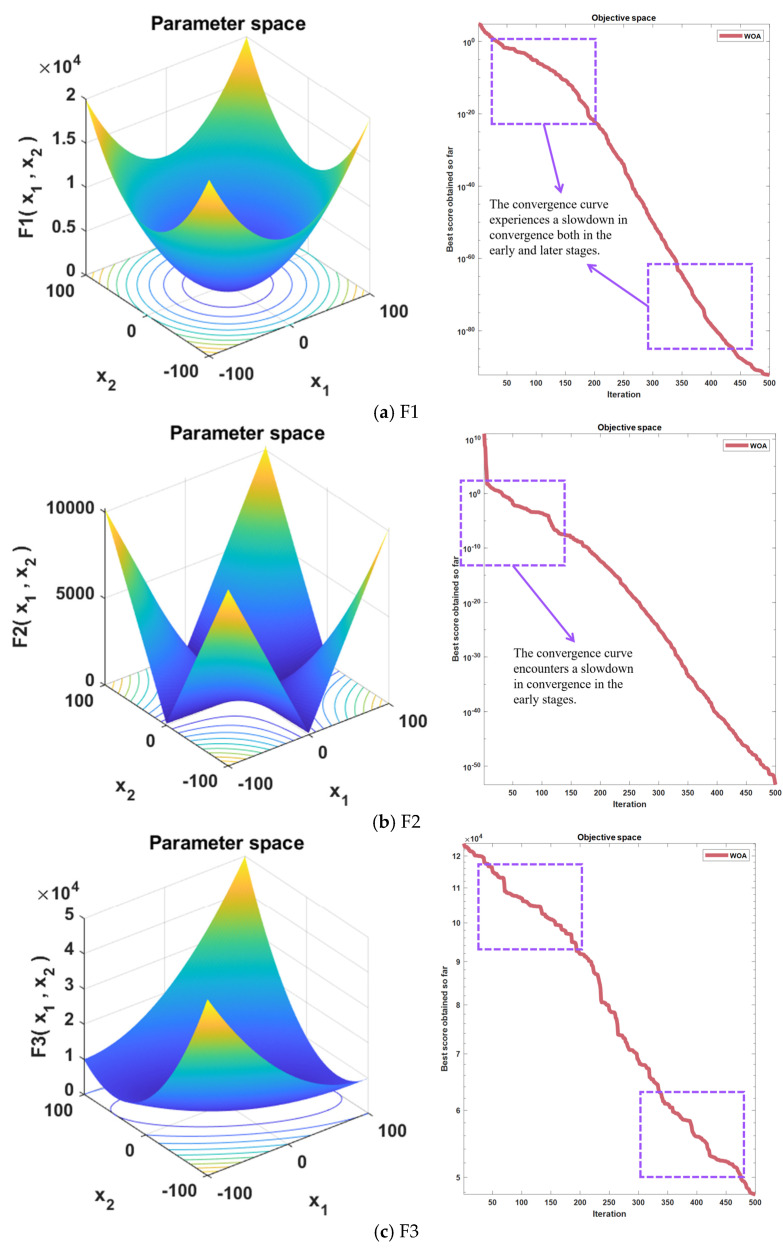

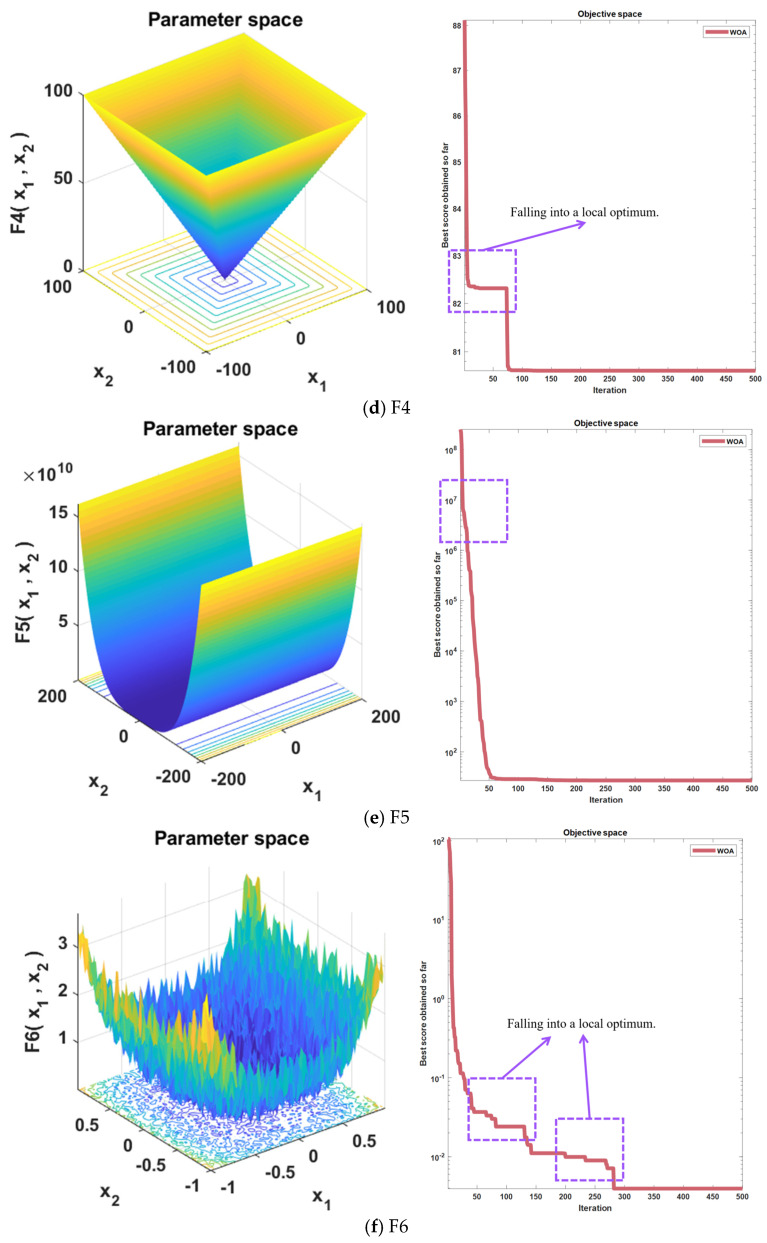
Function images of the 6 test functions with the corresponding algorithmic convergence plots.

As depicted in Figure 2, the Whale Optimization Algorithm (WOA) exhibits a deceleration in convergence during both the initial and final phases. This phenomenon is detrimental to the algorithm’s capability for global exploration and local convergence, thereby adversely affecting the ultimate efficiency and accuracy of the algorithm. On the other hand, the WOA is susceptible to the influence of local optima in the early stages, leading to a decline in algorithmic precision.

#### 2.2.2. Convergence Dynamics: Optimizing the Factor and Compensation Mechanism

In accordance with the analysis presented in Section 2.2.1, to more effectively address issues such as inadequate global convergence capabilities and a propensity to fall into local optima, it is imperative to refine the mathematical model underlying the algorithm.

**Figure 3:**
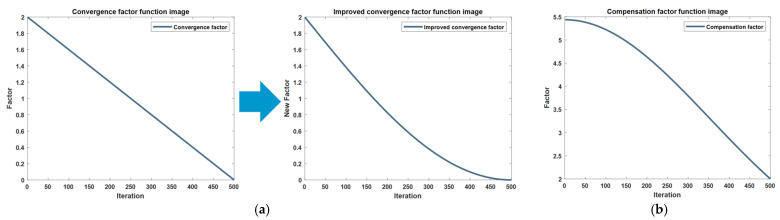
Functional image of factor (**a**) improvement of convergence factors; (**b**) compensation factor.

Within the framework of prey encirclement and bubble net assault, as articulated by Equation (4), it is imperative to recognize that individual whales amend their positional coordinates via the vector ‘A’ throughout the procedure. The modulation of vector ‘A’ exerts a profound influence on the algorithm’s capacity for global versus local exploration. Simultaneously, the coefficient vector ‘A’ hinges on the convergence factor ‘a’, which is characterized by a linear decrement from 2 to 0, as depicted in Figure 3a. To bolster the algorithm’s global search efficacy at inception and its local convergence precision at culmination, identifying an optimal convergence factor is paramount. This factor should manifest a robust value in the nascent iterations and a diminutive value in the terminal stages. However, a linearly descending factor does not align with the algorithm’s global and local search proficiencies. Conversely, the factor must evolve in tandem with the increment of iterations. Drawing from the extant literature [24], an adaptive factor $t⁄t_{max}$ is introduced, which is designed to be responsive to iterative fluctuations. Post the integration of this adaptive factor, the refined nonlinear convergence factor is articulated as presented hereinafter:

(6) $$a=2-2\cos\left[\frac{1}{2}\left(1-\frac{t}{t_{\max}}\right)\pi \right]$$

The functional image of the original convergence factor and the functional image of the improved convergence factor are shown below:

The revised convergence factor is intentionally calibrated to manifest reduced magnitudes during the intermediate and terminal phases of iteration, aligning seamlessly with the need for enhanced global search efficacy in the algorithm’s latter stages. This refined iteration of the Whale Optimization Algorithm (WOA) has been designated as Basic Version one Whale Optimization Algorithm (B1WOA). A comparative analysis of the convergence trajectories of B1WOA in juxtaposition with the pristine WOA across a suite of six benchmark test functions—the same set to be leveraged for all subsequent comparative evaluations—is delineated in the ensuing graphical representation.

**Figure 4:**
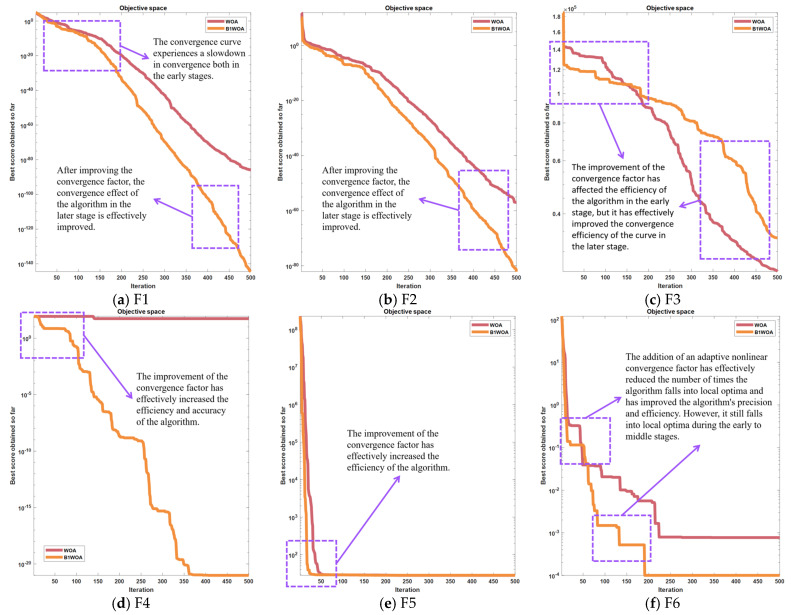
Convergence plots for 2 algorithms out of 6 test functions.

Figure 4 provides a comparative analysis demonstrating that for instances (a) and (b), the divergence between B1WOA and the original WOA escalates as the iteration count surpasses 150. Notably, B1WOA achieves a higher order of final convergence precision than the WOA. In (d), B1WOA outperforms the WOA in optimization efficacy. In (e), while both algorithms exhibit similar levels of accuracy, B1WOA is characterized by a more expeditious convergence velocity. The convergence curves depicted in Figure 4f suggest that both methodologies are adept at averting entrapment in local optima; however, it is B1WOA’s augmented global search prowess that confers its superior performance. In terms of convergence precision, B1WOA is observed to encapsulate a subset of the solution space of the WOA. Figure 4c indicates that B1WOA experiences a precipitous initial decline in convergence ratio, culminating in a final precision that falls short of the WOA’s.

As demonstrated by the outcomes presented in Figure 4, an analysis was conducted based on the linearity of the convergence curve. Following the incorporation of an adaptive convergence factor, the algorithm’s curve exhibited a marked alteration in the later stages of iteration, effectively ameliorating the issue of poor convergence efficacy in the latter phases of the algorithm. However, during the initial and intermediate phases, the premature flattening of the convergence factor led to a decline in the algorithm’s capability for global search, resulting in the premature flattening of the convergence curve and even entrapment in local optima as depicted in Figure 4a–d during the early stages. It was also observed that in (f), the frequency of the convergence curve falling into local optima decreased, yet the algorithm still frequently becomes entangled in local optima during the early and intermediate stages, leading to suboptimal convergence accuracy.

Upon thorough analysis, we have deduced that this phenomenon is attributable to the alteration of the convergence factor, which effectively ameliorates the drawbacks in the later stages of the algorithm but exhibits subpar performance during the early to intermediate phases. In the trade-off between global search capability and local convergence capability, the introduction of an adaptive convergence factor disrupts the original equilibrium. The incorporation of the new convergence factor significantly enhances the algorithm’s convergence capability in the later stages but concurrently results in a sluggish convergence rate in the early stages. To ameliorate this phenomenon, a variable gain compensation mechanism is introduced to compensate for the convergence capability during the initial stages, thereby achieving a balance between the early and later phases of the algorithm. According to the analytical results, the convergence rate is influenced by the enhanced global search capability. Consequently, an adaptive nonlinear compensation factor, denoted as ‘s’, is designed to mitigate the impact on the algorithm’s convergence rate. This compensation factor is incorporated into Equation (2.4), with the updated expression provided as follows:

(7) $$\left\{\begin{array}{c} X\left(t+1\right)=X^{*}\left(t\right)-A\cdot D\cdot s,p<0.5\\ X\left(t+1\right)=X^{*}\left(t\right)+s\cdot D\cdot e^{bl}\cdot \cos\left(2\pi l\right),p\geq 0.5\\ s=2e^{{\left(1-\frac{t}{t_{\max}}\right)}^{2}} \end{array}\right.$$

Following the incorporation of compensatory factors, the augmented Whale Optimization Algorithm (WOA), henceforth referred to as Basic Version two Whale Optimization Algorithm (B2WOA), was juxtaposed with its progenitor, the original WOA, as well as its immediate predecessor, B1WOA, across a suite of six benchmark test functions. The ensuing comparative analysis elucidates the performance profiles of these algorithms, as detailed in the subsequent exposition.

**Figure 5:**
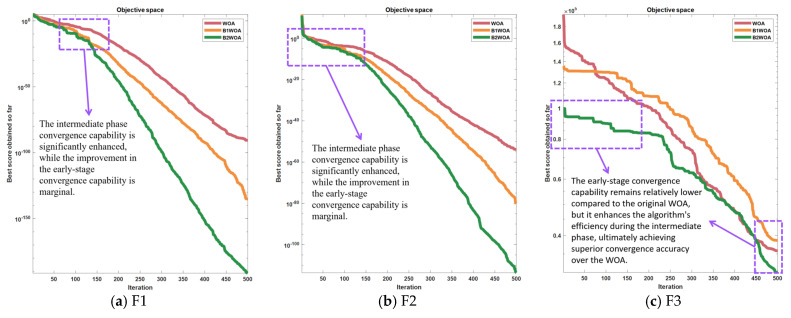

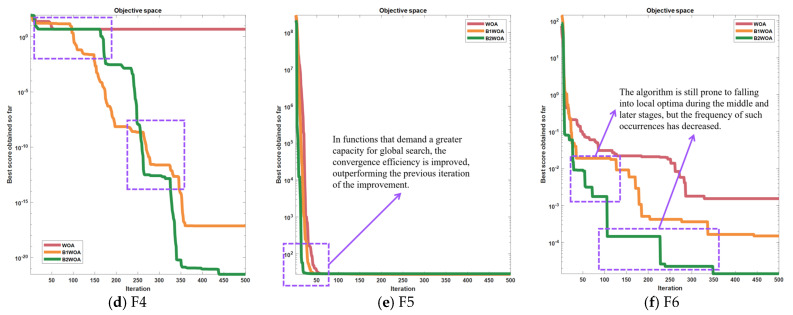
Convergence plots for 3 algorithms out of 6 test functions.

The incorporation of a variable gain compensation mechanism has effectively mitigated the impact of the new convergence factor on the algorithm’s convergence capability during the intermediate phase, as illustrated in Figure 5. In (d), the algorithm experiences a reduction in the incidence of becoming trapped in local optima, and there is a subsequent enhancement in accuracy. Nevertheless, the variable gain compensation mechanism affords minimal compensation for the convergence capability of the algorithm during the initial phase, resulting in a persistent issue of suboptimal convergence proficiency at the outset.

#### 2.2.3. Weight Adjustment: Balancing Exploration and Exploitation in the WOA

**Figure 6:**
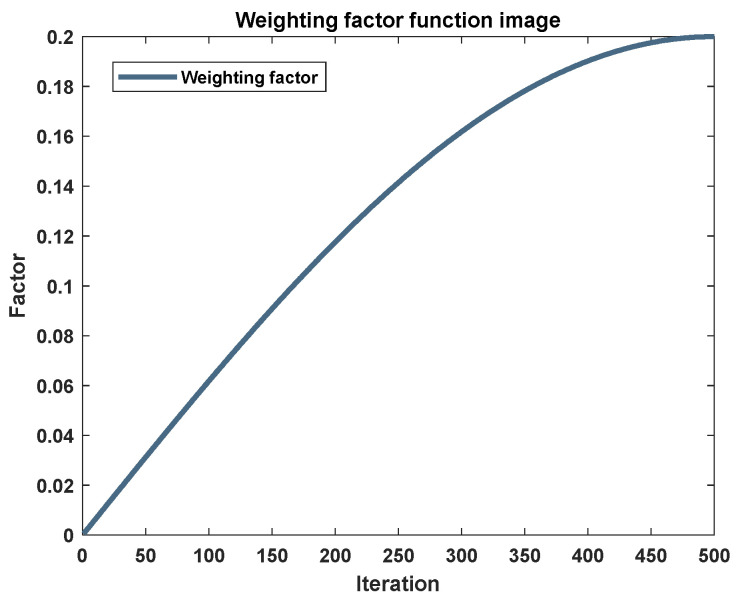
Image as a function of weight factor *ω*.

In light of the preceding analysis, it is evident that further refinement of the algorithm is warranted. Considering the compounded effects of the convergence factor and the variable gain compensation mechanism, it is imperative to introduce an adaptive weighting factor into the updated formula. The incorporation of this weighting factor can further influence the performance balance of the algorithm at various stages. By means of the weighting factor, an adaptive compensation of the convergence factor and convergence capability can be realized. The impact of the optimal individual on the update of positions in Equation (4) underscores the rationale behind the introduction of a weighting factor, denoted as ‘*ω*’. This factor is meticulously crafted to counteract the algorithm’s inherent deficiency in maintaining momentum during its approach to the optimal solution. Furthermore, acknowledging the algorithm’s reliance on robust global search capabilities in its initial phases, we have conceptualized the weight factor as a nonlinear adaptive element. This factor is intentionally configured to be minimal in the nascent iterations, gradually increasing to a more substantial magnitude in the later stages, as depicted in Figure 6. The mathematical formulation of this factor is presented as follows:

(8) $$\omega =\frac{1}{5}\cos\left[\frac{1}{2}\left(1-\frac{t}{t_{\max}}\right)\pi \right]$$

The judicious introduction of a weighting factor into the algorithmic framework effectively modulates the influence exerted by the optimal whale’s positional vector, $X^{*}\left(t\right)$, on the iterative adjustment of individual positions. This nuanced approach is pivotal in augmenting the algorithm’s capacity for extensive global foraging at the commencement of the search process. In stark contrast, as the algorithm progresses through an increasing number of iterations, the optimal whale’s positional vector $X^{*}\left(t\right)$ wields a more pronounced influence within the group. This gradual enhancement of the optimal position’s impact expedites the convergence of the remaining population, thereby accelerating the overall convergence velocity of the algorithmic ensemble.

Upon the integration of the weighting factor into the Whale Optimization Algorithm (WOA), yielding an optimized variant termed Test Weighted Whale Optimization Algorithm (TWOA), a comparative analysis was conducted. This analysis juxtaposed the performance of TWOA against its progenitors: the original WOA, the enhanced B1WOA, and the subsequent B2WOA. The comparison spanned across a diverse set of six distinct test functions designed to evaluate the efficacy of each algorithmic iteration.

**Figure 7:**
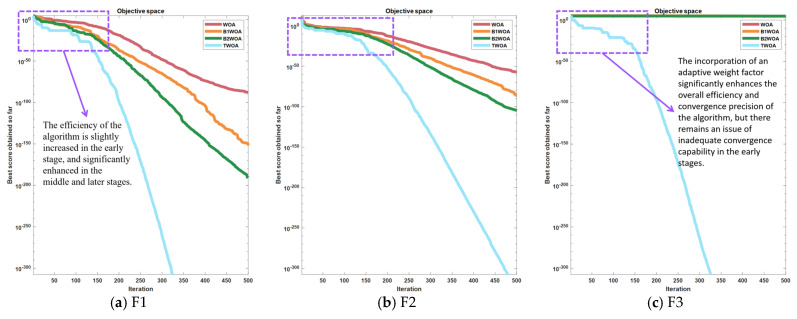

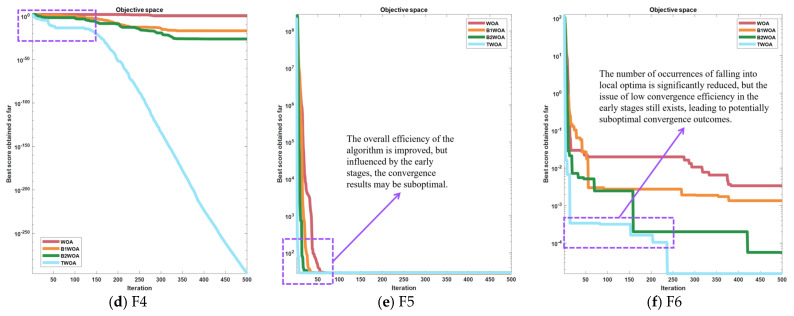
Convergence plots for 4 algorithms out of 6 test functions.

An examination of Figure 7 discloses a pronounced transformation in the convergence trajectories for instances (a) and (b), accompanied by a significant refinement in the precision of convergence. In scenario (c), the integration of the weighting factor serves to amplify the algorithm’s capacity for global exploration and the velocity of convergence, culminating in a marked enhancement in TWOA’s efficacy when juxtaposed with its three counterparts. This augmentation translates to a substantial advancement in accuracy for TWOA in Test Function 3, surpassing the performance of the other algorithms. In the context of (d), TWOA demonstrates a substantial improvement in its capacity to evade local optima, endowing it with a distinct advantage in accuracy over the trio of algorithms. Despite this, no significant disparities are observed between TWOA and the other algorithms in this regard. Nevertheless, TWOA manifests a considerable acceleration in convergence velocity, curtailing the temporal expanse requisite for the algorithm to attain an optimal solution. Additionally, TWOA is characterized by a markedly reduced frequency of local optima occurrences compared to its competitors, as illustrated in (f). The convergence curve’s configuration for TWOA is distinctly divergent from the others, and ultimately, TWOA proves to be more precise in its computations.

### 2.3. Macroscopic Enhancement: Advancing the WOA Through Strategic Insights

From a macroscopic vantage point, the ascent of a whale from the sea floor is characterized by an irregularly modulated trajectory, not subject to uniform contraction. The humpback whale, in particular, demonstrates a significant degree of agency over the extent of this contraction, which is contingent upon the spatial orientation of its prey. This biological observation is mirrored in the algorithmic construct, as articulated in Equation (2.3), illustrating the dynamic process of spiral indentation. Conversely, within a homogenous population of whales, there exists a collaborative effort to fulfill reconnaissance missions. Individual whales contribute to a unified endeavor in a manner that is synergistic and cohesive. This collective dynamic is algorithmically represented by the distribution of tasks across multiple whale groups, each designated to perform specific functions. To bolster the efficacy of the hunting strategy, it is imperative to enhance the prey’s search capability, thereby ensuring the overall hunting prowess of the whale population.

#### 2.3.1. Advanced Spiral Indentation: Enhancing the WOA’s Adaptive Maneuvering

Based on the analysis of the aforementioned results, both the convergence factor with a variable gain compensation mechanism and the adaptive weight factor have effectively improved the algorithm’s convergence accuracy and overall efficiency. However, the changes to the convergence factor and the addition of the compensation mechanism only significantly impact the middle and later stages of the algorithm, with minimal effects on the early stages. Therefore, it is necessary to analyze and improve the formulas involved in the search and convergence of the algorithm.

**Figure 8:**
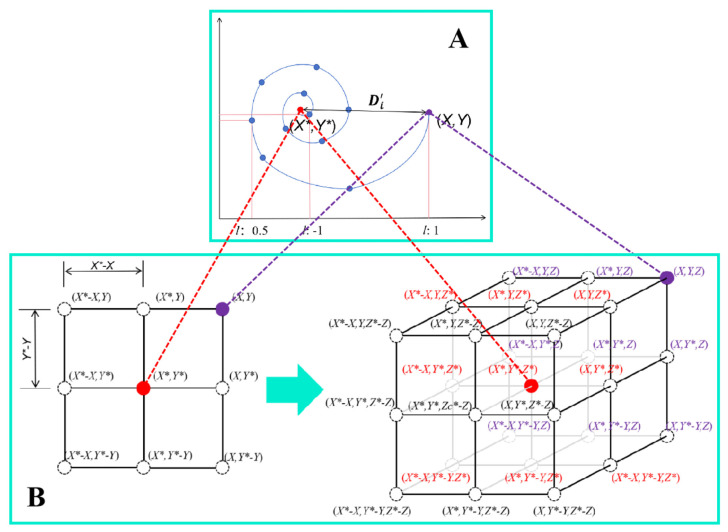
Spiral position updating and search space transformation. (**A**) the spiral convergence approach of whales within the search space (**B**) the transition of the search space from two dimensions to three dimensions.

Throughout the predation process, whales approach their prey along spiral trajectories characterized by distinct coefficients, which are contingent upon the spatial dynamics between the prey’s location and the whale’s instantaneous position. This adaptive maneuverability is facilitated by the continuous re-calibration of the whale’s spiral contraction amount, a phenomenon vividly illustrated in Figure 8. In (A), the spiral section reveals how the whale hunts in a spiral pattern; in (B), the left image demonstrates the basic principle of Equation (2) for solving two-dimensional problems, while the right image describes the potential update positions of search agents in three-dimensional space. It should be noted that by defining a random vector r, access to any point in the three-dimensional search space is possible. Consequently, the same concept can be extended to an n-dimensional search space, where search agents will move within the hypercube, circumscribing the best solution obtained thus far. It has been observed that in Equation (3), the spiral contraction amount is influenced by two factors: ‘b’ and ‘l’, whereas in the original WOA, only the alteration of ‘l’ is considered. A discussion and analysis of ‘b’ is undertaken to address the low-efficiency issues present in the early stages of the algorithm.

Within the algorithmic construct, the coefficients serve as fundamental constants, orchestrating the geometric delineation of the spiral’s trajectory. In the archetypal WOA, the spiral modulation coefficient, denoted as ‘b’, is uniformly initialized to a value of 1. During the iterative process of position refinement, the spiral indentation undergoes velocity-based modulation; yet, the spiral’s intrinsic geometric form persists unchanged. This inflexible mechanism, while systematic, encapsulates a limitation on the algorithm’s trajectory towards the prey, thus impeding its adaptive intelligence. In the natural habitat, humpback whales exhibit a dynamic adjustment of their indentation distance and locomotion, contingent upon the prey’s spatial orientation—a real-time adaptive strategy that is integral to their predatory maneuvers. By extracting inspiration from this organic paradigm, the enhancement of the algorithm’s heuristic acumen is advanced not by bestowing it with the innate cognitive faculties of a whale, but through the sophisticated refinement of the spiral indentation methodology. This strategic refinement aspires to replicate the adaptive agility observed in natural whale behaviors, thereby augmenting the algorithm’s capacity for intricate problem-solving and facilitating an advanced, nuanced traversal of the solution landscape.

The antecedent analysis has culminated in the conception of a novel spiral shape coefficient, designed to endow individuals within the algorithm with an expanded repertoire of traversal strategies, thereby augmenting the computational efficiency of the optimization process. Initially, the spiral’s geometric form is to be iteratively refined, allowing for the dynamic modulation of the whale’s approach distance relative to the prey. Consequently, this new spiral shape coefficient perpetuates the incorporation of an adaptive factor. Subsequently, the coefficient is crafted to enhance the local convergence of the algorithm, ensuring that it does not encroach upon the algorithm’s capacity for global exploration. Ultimately, the new spiral shape coefficient, forged in accordance with the aforementioned analytical principles, is articulated in the subsequent formulation:

(9) $$e^{b}=e^{7\cos\left[\left(1-\frac{t}{t_{\max}}\right)\pi \right]}$$

**Figure 9:**
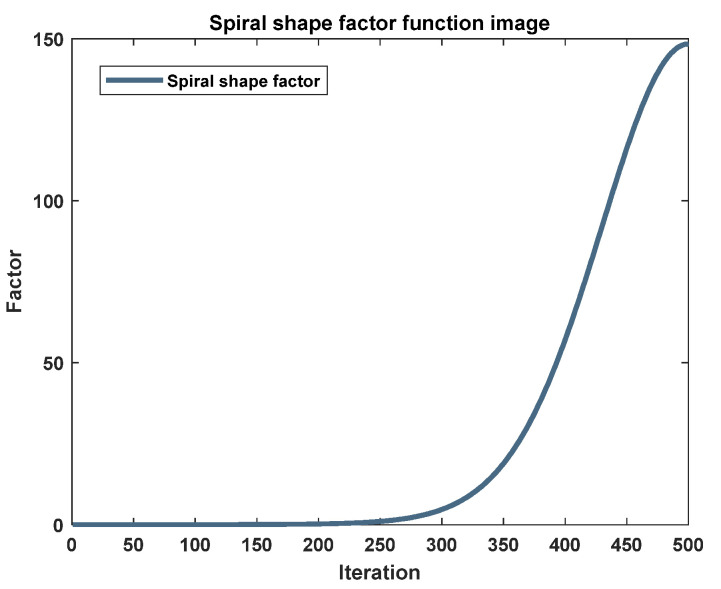
Image as a function of spiral shape factor.

The graphical representation of the novel spiral shape factor is illustrated in Figure 9. Observations reveal that this factor exhibits a positive correlation with the iteration count. Specifically, the spiral shape factor maintains a relatively stable value prior to the iteration threshold of 250, an attribute that facilitates a broad search for prey during the initial phase and bolsters the algorithm’s capacity to identify the optimal solution. Upon surpassing this iteration landmark, the solution undergoes global optimization. This is characterized by a marked escalation in the factor’s value, which amplifies the interval of the whale’s helix indentation, thereby abbreviating the timeframe to ensnare the optimal solution. Consequently, this amendment expedites the convergence velocity. The refinement of the spiral shape coefficient not only heightens the optimization precision and velocity but also elevates the overall efficacy of the algorithm.

**Figure 10:**
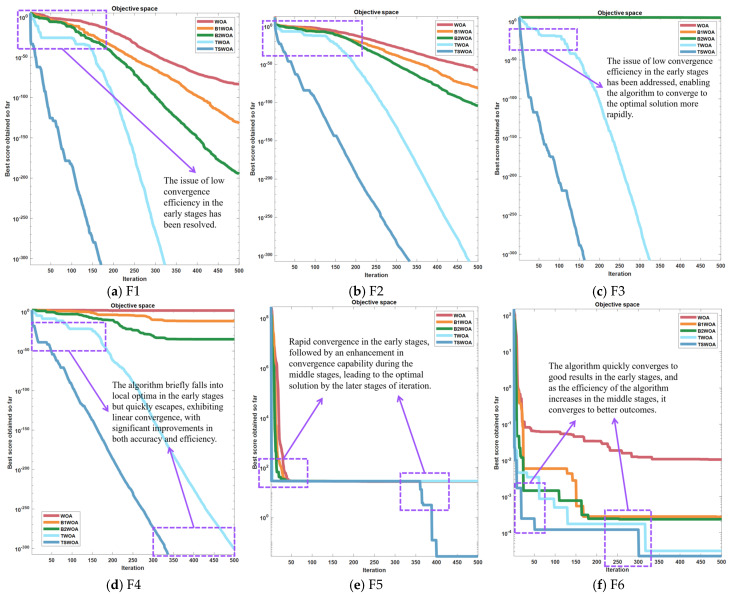
Convergence plots for 5 algorithms out of 6 test functions.

With the integration of the innovative spiral shape coefficients, the Test Improved Spiral Whale Optimization Algorithm, hereby designated as TSWOA, underwent a comparative evaluation against its foundational counterpart, the original WOA, alongside its evolved derivatives: B1WOA, B2WOA, and TWOA. This assessment spanned a curated selection of six test functions, meticulously crafted to provide a stringent examination of the efficacy of each algorithmic rendition. The comparative findings, which underscore the distinctive attributes and performance metrics of the algorithms, are schematically represented in Figure 10, presenting a graphical overview that encapsulates their comparative advantages and operational nuances.

As depicted in Figure 10, the convergence profiles of TSWOA in segments (a), (b), (c), and (d) exhibit more favorable line shapes compared to those of TWOA. Notably, a substantial acceleration in convergence velocity is observed. In segment (e), the refinement of the spiral shape coefficients engendered a marked increase in TSWOA’s convergence velocity, surpassing that of its counterparts and adeptly circumventing local optima during the later iterations. This advancement in precision has precipitated an enhancement in convergence accuracy. In segment (f), TSWOA’s convergence line shape, characterized by a scarcity of inflection points, suggests a diminished likelihood of succumbing to local optima. In stark contrast to other algorithms, TSWOA evinces a novel breakthrough in accuracy, particularly in the final stages of iteration, showcasing its proficiency in evading local optima, thereby achieving further convergence accuracy refinements. In Figure 10f, TSWOA’s line shape, with its significantly diminished number of inflection points relative to other algorithms, underscores a reduced propensity for entrapment in local optima. This observation corroborates the algorithm’s improved accuracy and the consequential amelioration in convergence precision. Despite these advancements, it remains apparent that the convergence accuracy between TSWOA and TWOA has not diverged significantly. This aspect will be the focal point of the subsequent phase of the optimization endeavor.

#### 2.3.2. Population Stratification: Role Assignment in the WOA’s Multi-Swarm Approach

Within a cetacean population, individuals are stratified into cohorts or subpopulations predicated on their designated tasks. The autonomous determination to engage in encircling prey or in pursuit of it is contingent upon the whales’ independent observations and evaluative judgments. This natural paradigm is mirrored in the algorithmic construct, where a whale population is stochastically generated, with each whale assigned an evaluative coefficient. This coefficient is pivotal in ascertaining the whale’s role in either the encirclement or the quest for prey. Consequently, the subpopulation is delineated into distinct operational tasks. In scenarios characterized by complexity and variability, the aggregate performance of the algorithm is anticipated to be substantially reinforced.

In light of the aforementioned analysis, a stochastic judging factor $D_{a}$ is introduced, delineated within the interval [0,1]. The assignment of tasks to each individual within the whale population is governed by the magnitude of $D_{a}$ at the time of its generation. Specifically, when the value of $D_{a}$ is greater than or equal to 0.5, the individual is allocated to the subpopulation responsible for the encirclement of prey. Conversely, when $D_{a}$ is less than 0.5, the individual is assigned to the subpopulation engaged in the pursuit of prey. This probabilistic allocation mechanism ensures a dynamic and adaptive distribution of roles within the population, enhancing the algorithm’s capacity to respond to the complexity and variability of the search space. When the two subpopulations operate separately, *ω* will be updated according to the following formula, aiming to enhance the connectivity and efficiency between each subpopulation:

(10) $$\left\{\begin{array}{c} \omega_{C\mathrm{apture}}=2D_{a}\cdot \omega \\ \omega_{\mathrm{Search}}=\frac{1}{2}D_{a}\cdot \omega \end{array}\right.$$

In Equation (6), *ω*_*Capture* and *ω*_*Search* represent the weights for the encircling process and the searching process, respectively, and are applied in Equation (8). The new Whale Optimization Algorithm conducts the search based on the aforementioned formulas.

The stochastic nature of population generation leaves the distribution of whales across the search space uncharted. Engaging in tasks assigned by randomly generated “task codes” facilitates systematic search and encirclement maneuvers. It is undeniable that certain whales, which are proximate to the optimal solution, may be reassigned to the search population by task allocation, potentially impeding the algorithm’s convergence process. However, in terms of the robustness of the algorithm, the introduction of a multi-population mechanism exerts a positive regulatory effect. Particularly within high-dimensional search spaces, the addition of a multi-population mechanism aids in enabling whales to conduct agile and swift searches within the complex, high-dimensional search space.

The improvement scheme articulated above paves the way for the genesis of an innovative variant of the whale optimization algorithm. The strategic integration of adaptive factors coupled with refinements to the spiral indentation methodology confers a marked enhancement in both global search and local convergence capacities. Moreover, the introduction of multi-swarm mechanisms serves to bolster the algorithm’s resilience against premature convergence, thereby fostering a more robust and efficient optimization process.

## 3. Multi-Swarm Improved Spiral Whale Optimization Algorithm (MISWOA): An Overview

### 3.1. Mathematical Modeling of the MISWOA: Enhanced Formulation and Parameters

Following the aforementioned enhancements, the enhanced whale optimization algorithm is designated as MISWOA. In Equations (1) and (2), the expression is updated as follows:

(11) $$\left\{\begin{array}{c} X\left(t+1\right)=\omega \cdot X^{*}\left(t\right)-A\cdot D\\ D=\left|C\cdot X^{*}\left(t\right)-X\left(t\right)\right| \end{array}\right.$$

(12) $$\left\{\begin{array}{c} A=2ar_{1}-a\\ a=2-2\cos\left[\frac{1}{2}\left(1-\frac{t}{t_{\max}}\right)\pi \right]\\ C=2r_{2} \end{array}\right.$$

In the aforementioned formula, *ω* represents two sets of weights, specifically $\omega_{s}$ for the search population and $\omega_{c}$ for the encirclement population, with the subsequent formulas following the same convention. In Equation (3), the expression is updated by adding weighting factors and improving the spiral indentation:

(13) $$\left\{\begin{array}{c} X\left(t+1\right)=\omega \cdot X^{*}\left(t\right)+D\cdot e^{7\cos\left[\left(1-\frac{t}{t_{\max}}\right)\pi \right]\cdot l}\cdot \cos\left(2\pi l\right)\\ D=\left|C\cdot X^{*}\left(t\right)-X\left(t\right)\right| \end{array}\right.$$

The original Equation (4) is updated as follows:

(14) $$\left\{\begin{array}{c} X\left(t+1\right)=X^{*}\left(t\right)-A\cdot D\cdot s,p<0.5\\ X\left(t+1\right)=X^{*}\left(t\right)+s\cdot D\cdot e^{7\cos\left[\left(1-\frac{t}{t_{\max}}\right)\pi \right]\cdot l}\cdot \cos\left(2\pi l\right),p\geq 0.5\\ s=2e^{{\left(1-\frac{t}{t_{\max}}\right)}^{2}} \end{array}\right.$$

In Equation (5), the expression is updated by adding the weighting factor:

(15) $$\left\{\begin{array}{c} X\left(t+1\right)=\omega \cdot X_{rand}\left(t\right)-A\cdot D\\ D=\left|C\cdot X_{rand}\left(t\right)-X\left(t\right)\right| \end{array}\right.$$

Based on the above analysis, the judging factor $D_{a}$ designed to be a random number of [0,1], and each individual chooses a different task according to the size of the judging factor when it is generated: when $D_{a}\geq 0.5$, the individual joins the subpopulation of rounding up the prey, and vice versa, when $D_{a}<0.5$, the individual joins the subpopulation of searching for the prey.

### 3.2. Flowchart Representation of the MISWOA: Visualizing the Algorithmic Process

**Figure 11:**
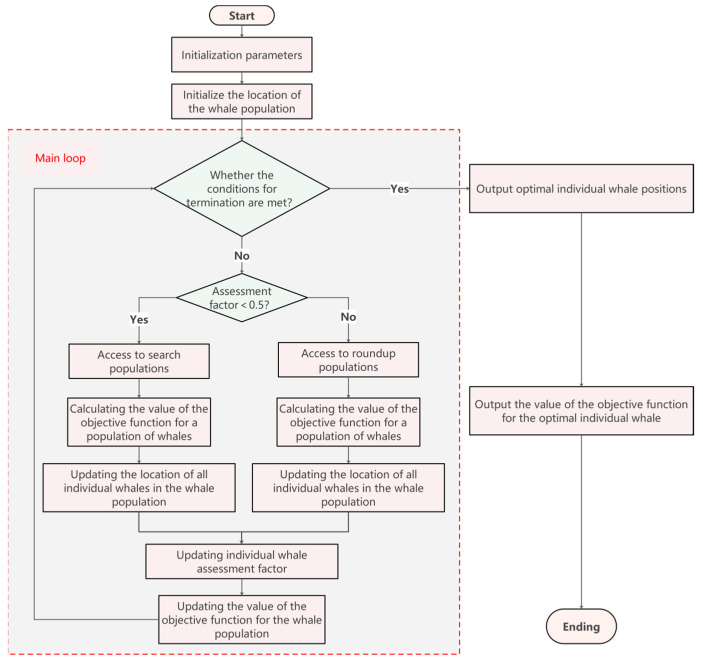
MISWOA Flowchart.

The enhanced Whale Optimization Algorithm, designated as MISWOA, is schematically represented in Figure 11. This algorithm initiates with a population that is stochastically generated and subsequently bifurcated into two distinct sub-populations. Further refinements to the algorithm are executed to ensure congruence with the authentic predatory strategies employed by humpback whales, achieved through the integration of an advanced strategic framework.

### 3.3. Pseudocode Implementation of the MISWOA: Detailed Operational Sequences

After delving into the theoretical underpinnings and pivotal attributes of the MISWOA, this segment endeavors to delineate the algorithm’s procedural specifics through the medium of pseudocode. As an abstracted programming construct, pseudocode peels back the layers to expose the algorithm’s logical scaffolding, circumventing the granular details of specific programming languages and rendering the algorithm’s foundational concepts and operational sequences with enhanced clarity.

The schematic flowchart presented in Algorithm 1 offers a panoramic visual depiction of the MISWOA, and the subsequent pseudocode transmutes this visual depiction into a series of meticulous operational directives. These pseudocodes are not only aligned with the discrete elements of the flowchart but also amplify the granularity of each procedural step, encompassing the initialization of parameters, the algorithmic rules for updating the positions of whales, and the conditions under which the algorithm concludes.
**Algorithm 1.** Pseudocode of MISWOA**% Algorithm Initialization**Initialize: Set the size of the whale population, the maximum number of iterations, and the algorithm parameters (including the convergence factor, compensation factor, weighting factor, etc.)for each whale i in the whale populationInitialize the position Xi of whale iEvaluate the fitness of whale iIdentify and record the global optimal position $X^{*}$
**% Begin Iteration****while** t < max_iter        **for** each whale i in the whale population                Update the convergence factor by the Equation (12)                Update the compensation factor by the Equation (7)                Update the weighting factor by the Equation (8)                Update the spiral shape factor by the Equation (9)                Calculate the new position based on the updated parameters                Check if the new position meets the conditions and update the position                **if** Meets Conditions(X_new) then                        Update the position of whale i to X_new
                **end if**

        **end for**

        **% Check if the global optimal position needs to be updated**
        Update Global Optimal Position $X^{*}$

        **% Assign tasks to the whale population based on the judging factor *D_a_***
        **for** each whale i in the whale population                $D_{a}$ = Rand                **$\mathrm{if}\;D_{a}\geq 0.5$** then                        Whale i joins the subpopulation responsible for encircling prey
                **else**
                        Whale i joins the subpopulation engaged in searching for prey
                **end if**

        **end for**
**        % Increment the iteration count**        t=t+1**end while****% Output the result**Output the global optimal position $X^{*}$ as the result of the algorithm

### 3.4. Algorithm Analysis: Considering Complexity from a Temporal Perspective

Time complexity can serve as an important measure of the computational effort required by an algorithm. In the WOA, let the scale of whales in the algorithm be N, the maximum number of iterations be T, and the problem dimension be D. The time complexity of the WOA can be expressed as O (N * T * D). The Multi-Swarm Improved Spiral Whale Optimization Algorithm (MISWOA) improves upon the original algorithm. From the algorithmic process, it can be seen that the inclusion of a convergence factor with an adaptive compensation mechanism, adaptive weights, and variable spiral update strategies does not increase the number of loops. Therefore, the time complexity remains O (N * T * D). The introduction of a multi-population mechanism does not increase the overall scale, number of iterations, or problem dimension of the algorithm. Overall, the time complexity of MISWOA is on par with that of the standard WOA.

## 4. Algorithm Efficiency Evaluation: Simulation Comparison and Experimental Verification

To assess the algorithmic efficiency of the MISWOA, three comparative analyses were conducted: a convergence comparison among each version of the algorithm throughout the improvement process, a convergence comparison between the MISWOA and both classical algorithms and other variants of the WOA, as well as an analysis of the operational efficiency of the MISWOA in engineering applications. To ensure the comparative effectiveness of the algorithms, the test functions used in this section are consistent with those used in previous chapters. Details of the functions can be found in Table 1.

### 4.1. Comparative Performance Evaluation of the MISWOA Against Benchmark Algorithms

The comparative evaluation of the Multi-Swarm Improved Spiral Whale Optimization Algorithm (MISWOA) alongside its foundational and variant counterparts—namely, the Base Whale Optimization Algorithm (WOA), the Base Version one Whale Optimization Algorithm (B1WOA), the Base Version two Whale Optimization Algorithm (B2WOA), the Test Weighted Whale Optimization Algorithm (TWOA), and the Test Improved Spiral Whale Optimization Algorithm (TSWOA)—was executed across a suite of six benchmark test functions. The ensuing outcomes of the convergence performance assessments are hereby presented.

**Figure 12:**
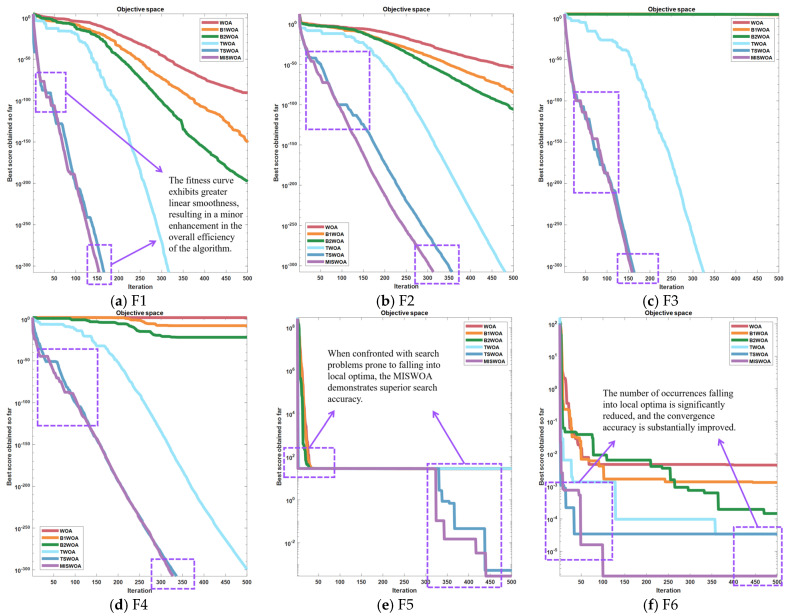
Convergence plots for 6 algorithms out of 6 test functions.

As illustrated in Figure 12, the Multi-Swarm Improved Spiral Whale Optimization Algorithm (MISWOA) demonstrates considerable superiority over other enhanced iterations across all evaluated dimensions. A comparison with the TSWOA reveals that the MISWOA’s fitness curves in segments (a), (b), (c), and (d) are more linearly smooth, indicating a marked improvement in convergence efficiency. When faced with functions such as (e) and (f) that are susceptible to getting trapped in local optima, the MISWOA utilizes the robustness of its multi-population mechanism to reliably converge towards more optimal solutions. Especially in the case of (f), the MISWOA exhibits outstanding performance, substantially increasing the accuracy of convergence.

### 4.2. Comparative Analysis of the MISWOA with WOA Variants and Other Optimization Algorithms

To conduct a comprehensive evaluation of the performance of the Multi-Swarm Improved Spiral Whale Optimization Algorithm (MISWOA), this study engages in a comparative analysis with other existing optimization algorithms, aiming to uncover its unique advantages and potential application domains. The foundational Whale Optimization Algorithm (WOA) and its variant, the Gravitational Search Whale Optimization Algorithm (GSWOA), are selected alongside other renowned optimization algorithms such as Particle Swarm Optimization (PSO), the Artificial Bee Colony Algorithm (ABC), and Grey Wolf Optimization (GWO) serving as benchmarks for this comparison. The comparison is based on multiple criteria, including convergence rate, solution accuracy, algorithmic stability, and the capability to search for multimodal functions. 

The ensuing test results will provide a detailed exposition of each algorithm’s performance on specific test functions and delve into the improvements of the MISWOA over other algorithms. Through this thorough comparative analysis, not only can we validate the efficacy of the MISWOA, but we can also offer guidance and insights for the future development of optimization algorithms.

**Figure 13:**
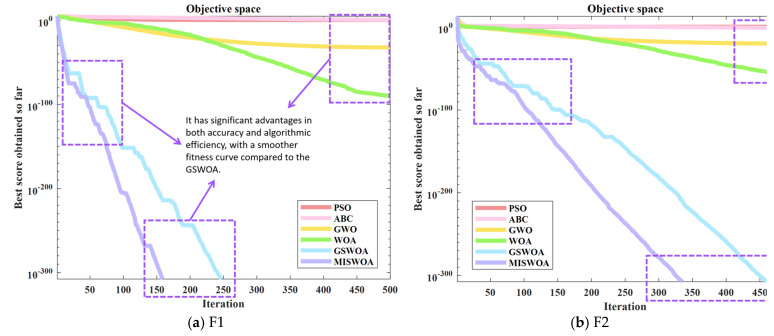

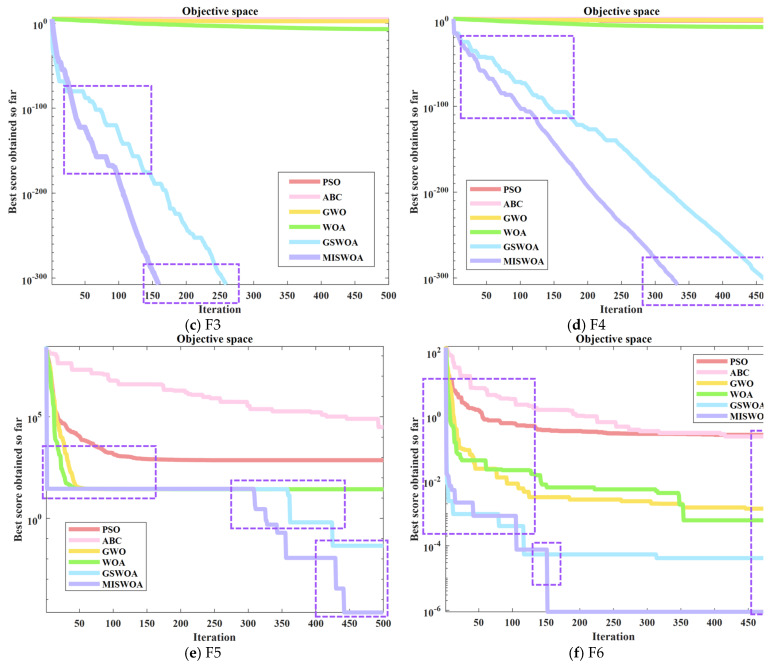
Convergence plots for 6 algorithms out of 6 test functions.

According to the content shown in Figure 13, the following conclusions can be reached: Firstly, in figures (a), (b), (c), and (d), the MISWOA, compared to the WOA, its variant GSWOA, and other exemplary algorithms, exhibits significant advantages in terms of convergence accuracy and rapidity. On the other hand, while the MISWOA may not match the exceptional algorithmic robustness of the PSO, GWO, and WOA, it demonstrates superior smoothness in the fitness curve compared to the GSWOA. Secondly, in figures (e) and (f), the MISWOA also leads other algorithms in terms of convergence velocity and precision. It is noteworthy that in figure (e), the MISWOA was once trapped in a local optimum for an extended period, escaping at around the 300th iteration, and then converging to a more optimal solution. This breakthrough from the local optimum corresponds to the spiral coefficient mentioned earlier, further validating the effectiveness of the improvement. The subsequent section will analyze the “maximum, minimum, mean, and standard deviation” recorded in the simulation, with the data recorded as shown in Table 2 below.

Based on the data recorded in Table 2, it is evident that both the GSWOA and MISWOA have performed exceptionally well. However, in tests F5 and F6, the MISWOA outperforms the GSWOA in terms of maximum and minimum values, which is particularly evident in the mean and standard deviation. At the same time, in the CEC 2017 tests (Appendix A, Table A1 and Table A2), MISWOA ranked first in 24 functions and second in 5 functions (details of the functions and test results are shown in the attachment).

Integrating the previous analysis of the fitness curves, it can be concluded that, by incrementally refining the shortcomings of the original WOA, the MISWOA distinguishes itself among various elite algorithms, significantly leading in terms of convergence speed and accuracy. Moreover, with its relatively superior robustness, it is capable of rapidly and precisely converging to the optimal solution in multidimensional and complex search spaces.

### 4.3. Algorithm Efficiency Analysis: The Application of MISWOA in Practical Engineering

The two aforementioned simulations elucidate two principal points: the efficacy of the algorithmic enhancements and the comparative excellence of the Multi-Swarm Improved Spiral Whale Optimization Algorithm (MISWOA) over other algorithms. However, there is often a considerable discrepancy between the outcomes of simulations and those of real-world applications. To address this issue, empirical validation is conducted to confirm the effective improvement of the MISWOA and to assess its potential in practical engineering applications. The experimental setup is as follows: the MISWOA is utilized for the automatic tuning of PID parameters to achieve control over an Automated Guided Vehicle (AGV). Subsequently, trajectory tracking experiments are conducted. It should be noted that PID control, a classical method in the field of control engineering, consists of proportional, integral, and derivative components. To optimize the PID control effect, it is necessary to adjust the three parameters: Kp, Ki, and Kd. Given the number of parameters that require tuning, the MISWOA is configured as three-dimensional in the experiment, with a population size of 50 and a maximum of 500 iterations. The experiment aims to validate the algorithm’s convergence accuracy and rapid response capability through trajectory tracking precision and positioning accuracy. Within the industry, trajectory tracking is typically maintained within a tolerance of ±5 mm, while positioning accuracy is controlled within ±3 mm. The efficacy of the controller is largely contingent upon the efficiency and accuracy of the optimization algorithm; hence, these two metrics are indicative of the algorithm’s practical performance.

The frequency of data collection for this experiment was 600 times per minute. The experimental setup is depicted in Figure 1, which includes the following components: data acquisition interface (A), map coordinates and angle explanation (B), visualization of target and actual speeds (C), an explanation of the experimental process (D), visualization of coordinates post data collection (E), and data visualization analysis results for velocity in the X-direction (F) and angle (G). The segments Position 2 (P2)–P3 and P5–P41 represent linear regions, while the segment P3–P5 corresponds to a Bezier curve region. The installation of the pre-installed platform has an error of 0.75°, which is also reflected in points P41 and P42. The velocity in the linear regions was set at 0.5 m/s, and in the curve regions, it was set at 0.3 m/s.

**Figure 14:**
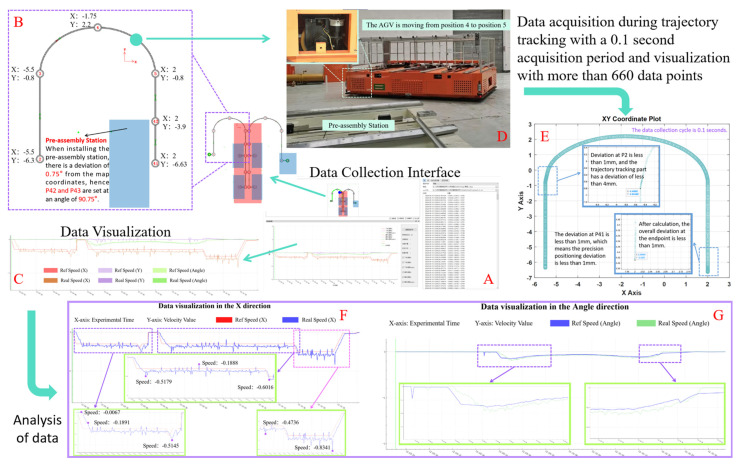
Experimental Design and Data Collection. (**A**) data acquisition interface (**B**) map coordinates and angle explanation (**C**) visualization of target and actual speeds (**D**) an explanation of the experimental process (**E**) visualization of coordinates post data collection (**F**) data visualization analysis results for velocity in the X-direction (**G**) data visualization analysis results for velocity in the angle.

According to the calculations based on the data from E, it is determined that in the trajectory tracking experiment, the tracking accuracy is maintained within 4 mm and the positioning accuracy is controlled within 2 mm. Additionally, from F and G, it can be observed that under steady conditions, the maximum error between the actual velocity and the target velocity is kept within 0.012 m per second. It should be noted that two significant latency fluctuations exist in F, which correspond to positions P3 and P5 in B. After rigorous analysis, the occurrence of these fluctuation points is attributed to the experimental site. Based on the analysis of these results and other data in Figure 14, it is evident that the MISWOA exhibits superior performance and plays a significant role with immense potential in practical engineering applications.

## 5. Conclusions

The present study introduces the Multi-Swarm Improved Spiral Whale Optimization Algorithm (MISWOA), an innovative optimization approach that significantly enhances the performance of the foundational Whale Optimization Algorithm (WOA). Through a comprehensive analysis and rigorous experimental validation, the MISWOA has demonstrated its superiority across a multitude of benchmark test functions, showcasing its exceptional convergence precision, speed, stability, and efficiency.

This study offers multifaceted contributions. Initially, addressing the deficiency in the algorithm’s late-stage search capability, the original convergence factor has been enhanced to an adaptive nonlinear factor, thereby suppressing the convergence process in the later stages of the algorithm. Subsequently, to counteract issues arising from the modified convergence factor, a variable gain compensation mechanism has been integrated into the convergence factor to compensate for the algorithm’s early-stage search capability. Then, by incorporating a variable gain weight factor, the algorithm’s search and convergence capabilities are further balanced throughout the entire search process. Additionally, the improved spiral convergence method, combined with a new spiral shape coefficient, endows the algorithm with enhanced intelligence and robustness. Lastly, the multi-population mechanism simulates the cooperative dynamics within natural whale pods, synergistically enhancing the algorithm’s effectiveness and robustness, enabling it to achieve efficient, excellent, and stable results even in multidimensional and complex search spaces. Comparative tests with the WOA, the variant GSWOA, and other outstanding algorithms demonstrate the MISWOA’s superior search accuracy, convergence speed, and robustness. On another note, in the trajectory tracking experiment for Automated Guided Vehicle (AGV), the MISWOA, with its formidable performance, further reduces tracking and positioning accuracy to within 4 mm and 2 mm, respectively. This substantiates the effectiveness of the WOA improvements and the powerful search capabilities of the MISWOA, while also highlighting its promising performance and substantial potential in practical engineering applications.

While the results are promising, there remains scope for further refinement. Future work will focus on fine-tuning the algorithm’s parameters to accommodate diverse optimization landscapes and on integrating additional adaptive strategies to enhance the algorithm’s robustness and applicability. The exploration of the MISWOA’s potential in real-world applications, such as engineering design, resource allocation, and scheduling problems, will also be pursued. The selection of 500 iterations for this study has been deliberate, with spiral shape coefficients tailored to this number. However, it is recognized that the convergence effect may vary with different iteration counts. Future research will focus on calibrating spiral shape coefficients for varying iteration counts to adapt to diverse optimization scenarios. Furthermore, the ongoing evolution and hybridization of the algorithm will ensure its adaptability to a broader spectrum of multidimensional optimization challenges. In conclusion, the strategic enhancements proposed in this paper have significantly advanced the WOA, yielding a more potent and versatile optimization algorithm in the MISWOA. The research not only validates the effectiveness of the proposed improvements but also sets the stage for future innovations in the field of computational intelligence and optimization.

## Figures and Tables

**Table 1:** Details of the test functions and parameter settings.

Function	Dim	Range	$f_{min}$
$f_{1}=\sum_{i=1}^{n}x_{i}^{2}$	30	[−100,100]	0
$f_{2}=\sum_{i=1}^{n}\left|x_{i}\right|+\prod_{i=1}^{n}\left|x_{i}\right|$	30	[−100,100]	0
$f_{3}=\sum_{i=1}^{n}{\left(\sum_{j=1}^{i}x_{i}\right)}^{2}$	30	[−100,100]	0
$f_{4}=max\left\{\left|x_{i}\right|,1\leq i\leq n\right\}$	30	[−100,100]	0
$f_{5}=\sum_{i=1}^{n}\left[100{\left(x_{i+1}-x_{i}^{2}\right)}^{2}+{\left(x_{i}-1\right)}^{2}\right]$	30	[−30,30]	0
$f_{6}=\sum_{i=1}^{n}{ix}_{i}^{4}+random\left[0,1\right]$	30	[−1.28,1.28]	0

**Table 2:** Data records in testing.

**Function**	PSO				ABC			
	***Max***	*Min*	*Ave*	*std*	*Max*	*Min*	*Ave*	*std*
F1	7.4599	1.8545	3.5219	2.3471	3.5189	2.4263	2.907	0.44381
F2	8.2926	4.9234	7.2488	1.4305	0.31759	0.10948	0.16742	0.085598
F3	43,395.1142	34,886.2018	39,428.4801	3021.9169	35,288.2847	25,975.7478	30,005.8065	3641.7049
F4	62.9905	0.56234	28.5592	23.9493	51.6266	44.7209	48.2942	2.8149
F5	864.5574	229.6079	578.1343	250.8741	90,734.2456	11,395.4507	40,397.4394	29,910.2449
F6	0.83274	0.16729	0.36663	0.26503	0.23641	0.11773	0.17893	0.045389
**Function**	**GWO**				**WOA**			
	***Max***	***Min***	***Ave***	***std***	***Max***	***Min***	***Ave***	***std***
F1	$2.398{\;\times \;10}^{-33}$	$2.744{\;\times \;10}^{-34}$	$1.051{\;\times \;10}^{-33}$	$9.679{\;\times \;10}^{-34}$	$2.276{\;\times \;10}^{-84}$	$6.538{\;\times \;10}^{-93}$	$5.080{\;\times \;10}^{-85}$	$9.946{\;\times \;10}^{-85}$
F2	$1.602{\;\times \;10}^{-19}$	$2.582{\;\times \;10}^{-20}$	$7.740{\;\times \;10}^{-20}$	$5.674{\;\times \;10}^{-20}$	$2.625{\;\times \;10}^{-53}$	$1.806{\;\times \;10}^{-58}$	$5.353{\;\times \;10}^{-54}$	$1.168{\;\times \;10}^{-53}$
F3	350.8386	64.6217	186.3149	113.7272	$1.13{\;\times \;10}^{-7}$	$5.52{\;\times \;10}^{-10}$	$2.97{\;\times \;10}^{-8}$	$4.72{\;\times \;10}^{-8}$
F4	5.5611	2.5624	4.3056	1.095	$5.68{\;\times \;10}^{-8}$	$1.71{\;\times \;10}^{-8}$	$3.41{\;\times \;10}^{-8}$	$2.06{\;\times \;10}^{-8}$
F5	28.5296	25.1138	26.8241	1.2679	27.7911	27.2203	27.5688	0.23881
F6	0.0027234	0.00027989	0.0012787	0.00091023	0.0059078	0.00060328	0.0026484	0.0026208
**Function**	**GSWOA**				**MISWOA**			
	***Max***	***Min***	***Ave***	***std***	***Max***	***Min***	***Ave***	***std***
F1	0	0	0	0	0	0	0	0
F2	0	0	0	0	0	0	0	0
F3	0	0	0	0	0	0	0	0
F4	0	0	0	0	0	0	0	0
F5	0.044643	0.001179	0.010594	0.019046	0.025664	$2.3{\;\times \;10}^{-5}$	0.010114	0.010915
F6	0.0001186	$1.11{\;\times \;10}^{-6}$	$5.81{\;\times \;10}^{-5}$	$5.12{\;\times \;10}^{-5}$	$6.72{\;\times \;10}^{-5}$	$8.99{\;\times \;10}^{-7}$	$3.03{\;\times \;10}^{-5}$	$2.64{\;\times \;10}^{-5}$

## Data Availability

The data that support the findings of this study are available from the corresponding author, upon reasonable request.

## Appendix A

This content pertains to the functional details and test results of the CEC 2017 test suite.

**Table A1:** Details of the CEC 2017.

	No.	Functions	$F_{i}^{*}=F_{i\left(x^{*}\right)}$
Unimodal functions	1	Shifted and Rotated Bent Cigar Function	100
	2	—	—
	3	Shifted and Rotated Zakharov Function	300
Simple multimodal functions	4	Shifted and Rotated Rosenbrock’s Function	400
	5	Shifted and Rotated Rastrigin’s Function	500
	6	Shifted and Rotated Expanded Scaffer’s F6 Function	600
	7	Shifted and Rotated Lunacek Bi_Rastrigin Function	700
	8	Shifted and Rotated Non-Continuous Rastrigin’s Function	800
	9	Shifted and Rotated Levy Function	900
	10	Shifted and Rotated Schwefel’s Function	1000
Hybrid functions	11	Hybrid Function 1 (N = 3)	1100
	12	Hybrid Function 2 (N = 3)	1200
	13	Hybrid Function 3 (N = 3)	1300
	14	Hybrid Function 4 (N = 4)	1400
	15	Hybrid Function 5 (N = 4)	1500
	16	Hybrid Function 6 (N = 4)	1600
	17	Hybrid Function 6 (N = 5)	1700
	18	Hybrid Function 6 (N = 5)	1800
	19	Hybrid Function 6 (N = 5)	1900
	20	Hybrid Function 6 (N = 6)	2000
Composition functions	21	Composition Function 1 (N = 3)	2100
	22	Composition Function 2 (N = 3)	2200
	23	Composition Function 3 (N = 4)	2300
	24	Composition Function 4 (N = 4)	2400
	25	Composition Function 5 (N = 5)	2500
	26	Composition Function 6 (N = 5)	2600
	27	Composition Function 7 (N = 6)	2700
	28	Composition Function 8 (N = 6)	2800
	29	Composition Function 9 (N = 3)	2900
	30	Composition Function 10 (N = 3)	3000

**Table A2:** Data records of CEC 2017 in testing.

**Function**	**PSO**		ABC		GWO	
	***Ave***	***std***	*Ave*	*std*	*Ave*	*std*
F1	$8.59{\;\times \;10}^{8}$	$6.61{\;\times \;10}^{8}$	5.98${\;\times \;10}^{5}$	4.51${\;\times \;10}^{5}$	7.94${\;\times \;10}^{5}$	5.31${\;\times \;10}^{5}$
F3	8715.00	5223.00	15.00	10.20	600.00	709.00
F4	86.30	56.60	11.20	17.90	44.90	29.30
F5	48.10	7.50	30.30	6.32	44.20	11.50
F6	29.76	14.80	5.48	2.56	34.80	10.70
F7	97.11	14.10	48.20	5.37	43.20	27.10
F8	55.43	8.13	23.50	5.63	28.80	4.79
F9	239.10	140.00	2.54	2.50	120.00	68.30
F10	1100.00	235.00	1400.50	149.20	864.40	227.20
F11	286.00	121.00	36.20	21.90	36.20	21.90
F12	$7.36{\;\times \;10}^{6}$	$5.20{\;\times \;10}^{6}$	2.69${\;\times \;10}^{5}$	2.62${\;\times \;10}^{5}$	5.26${\;\times \;10}^{4}$	1.31${\;\times \;10}^{4}$
F13	$7.46{\;\times \;10}^{4}$	$6.05{\;\times \;10}^{4}$	8890	3480	1.03${\;\times \;10}^{4}$	7470.00
F14	471.00	630.00	59.70	11.40	80.50	58.40
F15	6380.00	3260.00	254.00	190.00	2110.00	1940.00
F16	143.00	77.30	120.20	58.10	76.40	46.70
F17	95.70	17.70	65.20	7.39	65.60	7.38
F18	$9.77{\;\times \;10}^{4}$	$8.29{\;\times \;10}^{4}$	4630.00	4390.00	1.20${\;\times \;10}^{4}$	2650.00
F19	8060.40	$1.70{\;\times \;10}^{4}$	84.60	83.50	615.20	274.60
F20	101.10	17.60	105.00	25.90	169.00	38.00
F21	246.00	29.30	118.00	19.70	211.00	43.70
F22	157.00	18.20	108.00	2.62	115.00	5.75
F23	391.00	30.10	323.00	7.65	331.00	9.13
F24	382.00	10.20	277.00	91.30	380.00	20.90
F25	470.00	30.50	434.00	20.50	447.00	1.82
F26	447.00	22.10	324.00	49.10	962.00	541.00
F27	452.00	27.40	396.00	3.56	414.00	13.70
F28	444.00	5.94	504.00	126.00	834.00	154.00
F29	289.00	30.50	314.00	18.40	335.00	47.90
F30	$3.32{\;\times \;10}^{6}$	$2.95{\;\times \;10}^{6}$	4.38${\;\times \;10}^{5}$	6.85${\;\times \;10}^{5}$	3.73${\;\times \;10}^{5}$	3.10${\;\times \;10}^{5}$
**Function**	**WOA**		**GSWOA**		**MISWOA**	
	***Ave***	***std***	***Ave***	***std***	***Ave***	***std***
F1	$1.07{\;\times \;10}^{6}$	$1.33{\;\times \;10}^{6}$	8140	4390	1780	1850
F3	402.00	258.00	0.01	0.03	0	0
F4	74.30	1.74	5.67	1.81	2.71	1.69
F5	60.40	20.70	22.60	7.76	19.60	8.11
F6	37.50	11.50	1.06	1.45	0.06	0.16
F7	87.20	26.20	31.20	8.94	31.10	7.44
F8	69.80	13.90	23.60	9.33	21.30	6.95
F9	699.00	388.00	1.32	2.96	0.04	0.25
F10	1020.00	408.00	794.00	312.00	679.00	257.00
F11	91.90	79.50	34.50	25.60	19.00	20.90
F12	$2.18{\;\times \;10}^{5}$	$1.32{\;\times \;10}^{5}$	2.46${\;\times \;10}^{4}$	8300	4580.00	4230.00
F13	$1.08{\;\times \;10}^{4}$	3630.00	1500.00	1150.00	908.00	552.00
F14	153.00	132.00	70.70	33.10	58.60	13.20
F15	4540.00	2630.00	216.00	130.00	173.00	68.40
F16	307.00	120.00	40.20	24.90	92.90	77.20
F17	48.40	13.10	124.00	81.10	52.40	14.20
F18	1570.00	4870.00	2010.00	1950.00	175.00	111.00
F19	$3.27{\;\times \;10}^{4}$	$2.87{\;\times \;10}^{4}$	107.00	65.30	74.00	44.30
F20	106.00	28.20	30.80	16.10	58.90	22.90
F21	219.00	61.50	182.00	55.80	198.00	46.10
F22	117.00	8.03	104.00	2.11	103.00	0.92
F23	347.00	22.20	325.00	7.61	322.00	7.40
F24	374.00	19.70	357.00	75.30	324.00	7.98
F25	424.00	14.20	430.00	22.40	422.00	23.60
F26	427.00	53.60	378.00	35.90	311.00	23.60
F27	411.00	15.30	394.00	2.99	393.00	2.78
F28	414.00	10.10	518.00	99.20	323.00	42.00
F29	311.00	32.30	313.00	49.50	263.00	23.90
F30	$3.83{\;\times \;10}^{6}$	$2.31{\;\times \;10}^{6}$	2.49${\;\times \;10}^{5}$	5.39${\;\times \;10}^{5}$	5.15${\;\times \;10}^{4}$	2.43${\;\times \;10}^{5}$
